# Energy and Arginine Density in the Diets of Arbor Acre Hens from 40 to 50 Weeks of Age: Effects on Development and Lipid Metabolism of Embryos

**DOI:** 10.3390/ani13233737

**Published:** 2023-12-03

**Authors:** Zhongyu Wang, Jiawei Feng, Chunxu Yang, Shaoyang Mou, Yingjie Xie, Xiaoxue Duan, Zhongyu Li, Zhongpeng Bi, Tianshu Liu, Feng Li, Liangmei Xu

**Affiliations:** 1 College of Animal Science and Technology, Northeast Agricultural University, Harbin 150030, China; wzywzy19991001@163.com (Z.W.); 18509555518@163.com (J.F.); yangcx026@163.com (C.Y.); xieyingjie1105@163.com (Y.X.); sweetyduan@163.com (X.D.); potato_li@163.com (Z.L.); bnm0722@163.com (Z.B.); ltslts0906@163.com (T.L.); 2 Shandong Yisheng Livestock & Poultry Breeding Co., Ltd., Yantai 265508, China; moushaoyang@126.com

**Keywords:** arginine, energy, embryo, maternal effect, hormones, lipid metabolism

## Abstract

**Simple Summary:**

Fat accumulation not only leads to maternal health problems, but also to a decline in egg production and egg quality. Supplementing arginine in the diet or reducing the energy level can prevent fat accumulation. However, it is not clear whether supplementing arginine to hens and restricting diet will affect the lipid metabolism of the offspring. This experiment investigated just that. The results revealed that maternal restricted feeding improved embryonic development increased breast rate of embryos at E21, thigh rate of embryos at E19, and liver rate of embryos at E11 or E17. It also regulated lipid metabolism-related indices in embryonic serum: serum cholesterol content of embryos at E19, serum low-density lipoprotein content of embryos at E21, and serum glucose content of embryos at E13 were higher. Serum high-density lipoprotein content of embryos at E17, serum triglyceride content of embryos at E19 or E21, and serum nitric oxide synthases content of embryos at E11 and E19 were lower. Maternal dietary addition of digestible arginine had a significant effect on lipid metabolism indices in embryos: serum cholesterol content of embryos at E11 and serum glucose content of embryos at E11 were lower. Urea nitrogen in allantoic fluid was deceased from E11 to E15. Serum nitric oxide synthases content of embryos was higher from E11 to E19.

**Abstract:**

The effects of maternal dietary energy and arginine level on embryonic development and serum lipid metabolism were investigated in this study. A 2 × 3 factorial experiment was conducted with six treatments represented by 10 replicates of eight Arbor Acre broiler breeder hens each. Diets fed from 40 to 50 weeks of age were formulated to contain two digestible arginine levels (9.6 g/kg and 14.5 g/kg) and three metabolic energy levels (10.08 MJ ME/kg, 11.88 MJ ME/kg, and 13.68 MJ ME/kg). Artificial insemination was used, and eggs collected from 50 weeks of hens’ age were hatched. Embryonic growth, biochemical and endocrine indexes of embryonic serum and allantoic fluid were measured on different embryonic days (E). The results were as follows: Egg weight (E0, E11, E13) and embryonic weight (E12, E15) in the high-energy group (13.68 MJ ME/kg) were significantly decreased (*p* < 0.01), as were embryonic breast rate (E13, E15, E21), thigh rate (E13–E21) and liver rate (E15–E21). The reciprocal effects of arginine and energy were significant on breast rate (E11, E13, E17), thigh rate (E19, E21) and liver rate (E11, E19) of the embryo (*p* < 0.05). CHO (E13–E19), high-density lipoprotein (E13, E15, E21), low-density lipoprotein (E15, E19, E21), and blood glucose (E13) levels in embryonic serum decreased with the increase in maternal dietary energy level (*p* < 0.05), but triglyceride levels (E19, E21) showed the opposite result (*p* < 0.05). The levels of cholesterol and blood glucose in embryonic serum at E11 and urea nitrogen in allantoic fluid at E11–E15 were significantly decreased in the 14.5 g/kg arginine group (*p* < 0.01). With the increase in maternal dietary energy and arginine levels, embryonic serum nitric oxide synthases levels (E11, E15, E19) increased significantly (*p* < 0.01). The reciprocal effect of arginine and energy in maternal diets was significant on the embryonic serum high-density lipoprotein level at E21 (*p* < 0.05). Embryonic serum insulin levels at E13 were significantly elevated in the high-energy group (13.68 MJ ME/kg). The reciprocal effect of arginine and energy was significant on the embryonic serum growth hormone level (*p* < 0.01). Embryonic serum growth hormone levels were significantly reduced in the 14.5 g/kg arginine and 13.68 MJ/kg metabolic energy group (*p* < 0.01). In conclusion, maternal restricted feeding improved embryonic development and regulated lipid metabolism-related indices in embryonic serum. Maternal dietary addition of digestible arginine had a significant effect on lipid metabolism indices in embryos. There was a maternal effect of maternal dietary energy and arginine levels on embryo growth and development. The deposition of maternal nutrients affects the development of embryos.

## 1. Introduction

The growth and health of chicks is critical to the poultry economy, and several factors can affect the quality of chicks, such as the age of the breeder, the hatching method, storage time, etc. In addition, nutrition in eggs is one of the most important parameters affecting chicks [1]. Some studies have shown that eggs have an effect on the growth of chicks [2,3]. Embryonic development is a complex and coherent process, and it has been found that maternal diet influences the immunity and development of the offspring, with maternal nutrients being deposited into the egg and utilized by the offspring [4,5,6].

The energy standard for laying hens at onset of lay is 11.50 MJ/kg (NY/T33-2004 [7]). High-energy diets can significantly promote broiler growth (13.38 MJ/kg) [8]. In 5-week-old laying hens, dietary high energy (13.01 MJ/kg) can promote bone development and improve lipid metabolism [9]. However, it has been reported that broiler chickens would still be able to attain market weight when fed with a less-dense nutrient diet in the starter phase, followed by a high-energy diet in the finisher phase [10]. And, existing studies have found that restricted feeding increases egg production, average egg weight and hatchability in broiler breeders [11,12,13]. During the early and peak laying periods, fat synthesis and consumption in hens are in dynamic balance [14]. As the hen ages and liver function decreases, fat is deposited excessively, resulting in fatty liver hemorrhagic syndrome (FLHS). And not only that, but fat is denatured, which is one of the main causes of cardiovascular disease [15]. The increase in mortality in the late egg-laying period is closely related to lipid metabolism. The energy level in the diet determines the body weight of the hen during the laying period. Dietary restriction can solve the problem of excessive fat deposition in broilers and hens to a certain extent, and promote their growth and laying performance [16]. In contrast, high-energy diets not only increase feeding costs, but may also induce fat deposition and reduce egg production [17].

Arginine plays an important role in the energy metabolism and reproductive performance of birds, as an essential amino acid for birds [18,19]. In ovo provision of arginine regulates chick growth [20]. It was shown that the addition of arginine and guanidionoacetic acid to vegetable diets resulted in higher carcass weight and lower abdominal fat deposition in heat-stressed broilers compared to controls [21]. In addition, the addition of arginine and threonine (35 + 25 mg/egg) within the eggs improved feed intake and body weight of chicks after hatching compared to controls [22]. NO produced by arginine catabolism activates the AMPK pathway in vivo, enhances glycogen and lipid degradation, reduces lipid synthesis, and improves the body’s insulin sensitivity [23,24,25]. Supplementing L-arginine with a high-energy diet can help broiler breeders improve fat metabolism and reduce fat deposition by reducing the gene expression of fatty acid synthase in the liver and promoting the expression of cardiac carnitine lipid acyltransferase and hydroxyalkyl coenzyme dehydrogenase [26].

Many of the above studies have shown that supplementation with arginine and restriction of energy levels in animal diets can improve lipid metabolism, but few studies have explored the effects of maternal ration levels on embryo lipid metabolism. The hypothesis tested in this study was that maternal energy-restricted feeding or dietary supplementation with arginine would promote embryonic development and improve embryonic lipid metabolism. The energy and arginine standard for laying hens at onset of lay is 11.50 MJ/kg and 1.02 g/kg (NY/T33-2004). Therefore, according to the regulations, we set the maternal diet by setting three energy levels (10.08 MJ ME/kg, 11.88 MJ ME/kg, and 13.68 MJ ME/kg) and two arginine levels (9.6 g/kg and 14.5 g/kg). The effects of maternal energy levels or arginine levels on embryonic growth and lipid metabolism were investigated by measuring egg weight, eggshell weight, weight of embryo, breast rate of embryo, thigh rate of embryo, liver rate of embryo, serum cholesterol (CHO) content of embryo, serum high-density lipoprotein (HDL) content of embryo, serum low-density lipoprotein (LDL) content of embryo, serum triglyceride (TG) content of embryo, serum blood glucose (GLU) content of embryo, serum nitric oxide synthase (NOS) content of embryo, serum growth hormone (GH) levels of embryo, serum insulin (INS) levels of embryo, and level of urea nitrogen in allantoic fluid during the hatching period.

## 2. Materials and Methods

### 2.1. Birds and Experimental Design

This research was approved by the Animal Care and Use Committee of Northeast Agricultural University (NEAUEC20130202). The experiment was carried out in the animal experimental base of Xiangfang Farm, Northeast Agricultural University. Birds used in these experiments were cared for under the guidelines stated in the Guide for the Care and Use of Agricultural Animals in Agricultural Research and Teaching [27].

A 2 × 3 factorial design was used to evaluate the effects of maternal dietary energy and arginine levels. A total of 480 healthy Arbor Acre female broiler breeders at 40 weeks of age were randomly divided into six treatments (T1, T2, T3, T4, T5, and T6), with each treatment represented by 10 replicates of 8 birds each. T1 were feed a diet with 10.08 MJ/kg ME and 9.6 g/kg digestible arginine. T2 were fed a diet with 10.08 MJ/kg ME and 14.5 g/kg digestible arginine. T3 were fed a diet with 11.88 MJ/kg ME and 9.6 g/kg digestible arginine. T4 were fed a diet with 11.88 MJ/kg ME and 14.5 g/kg digestible arginine. T5 were fed a diet with 13.68 MJ/kg ME and 9.6 g/kg digestible arginine. T6 were fed a diet with 13.68 MJ/kg ME and 14.5 g/kg digestible arginine. Dietary composition and nutrition levels of broiler breeder hens during the laying period were shown in Table 1. The experimental diet was fed to hens from 41 weeks old for 10 weeks. At the age of 50 weeks, 150 eggs were collected from each group for artificial insemination and incubation. Serum and tissue samples were collected at 11, 13, 15, 17, 19 and 21 days of embryonic age.

### 2.2. Feeding and Management

**Figure 1:**
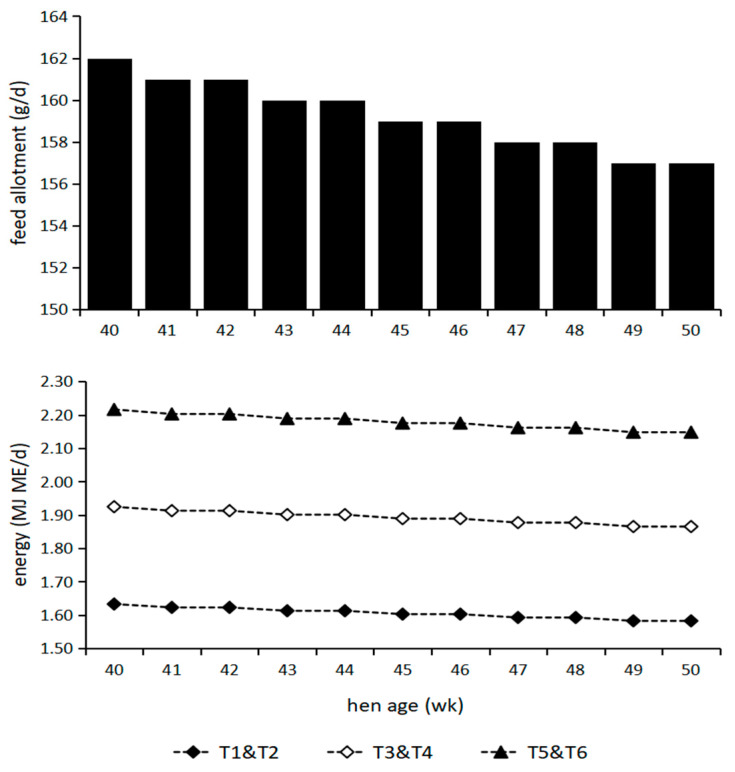
Daily feed and energy allotments provided for hens from 40 to 50 weeks.

The experimental period lasted 10 weeks from the end of 40 weeks. All treatments were restricted to feeding. The daily feed and energy allotments provided for hens from 40 to 50 weeks are shown in Figure 1. During the experiment, two birds each were housed in an individual cage (38 cm × 34 cm × 39 cm). Feed was given at 07:00 every day. There were no leftovers in the trough every day. Drinking water was ad libitum. Light was provided 16 h per day by combination of natural and artificial light. The room temperature was maintained at 20 ± 2 °C. The humidity was controlled at 55 ± 5%. Animal housing and handling procedures during the study were in accordance with guidelines of Handbook of Modern Broiler Production [29]. The experimental diet was fed to hens from 41 weeks old for 10 weeks. At the age of 50 weeks, 150 eggs produced during four consecutive days were collected from each group for artificial insemination (artificial insemination was performed twice every 5 days) and incubation and put into an automatic incubator (FT-ZF 10; Chun Ming Fang Tong Electronic Co., Ltd., Beijing, China) with their blunt tip upwards. These eggs did not include non-settable eggs such as misshapen eggs, dirty eggs, excessively large or small eggs, broken eggs, cracked eggs and eggs without a shell (but with intact membrane). Before hatching, the eggs were temporarily stored in a dark room with a temperature of 10~12 °C and relative humidity of 65~70%. Serum and tissue samples were collected at 11, 13, 15, 17, 19 and 21 days of embryonic age.

### 2.3. Data and Sample Collection

On embryonic day (E) 11, E13, E15, E17, E19 and the day of hatching (E21), two eggs per replicate from different cages were taken and weighed. The blunt end of each egg was opened. Embryonic serum samples on E11, E13, E15, E17, E19 and E21 were collected and kept in a −20 °C freezer as described by Niu et al. [30]. The embryonic weight and eggshell weight of each egg were recorded. The breast, thigh and liver were separated and weighed.

The breast rate, thigh rate and liver rate were calculated as follows:

organ index = (organ weight/embryo weight) × 100%.

Allantoic fluid was extracted from each embryonic egg on E11, E13, E15 and E17, and separated into EP tubes and stored at −20 °C.

Total cholesterol (CHO), low-density lipoprotein (LDL), high-density lipoprotein (HDL), triglyceride (TG) and glucose (GLU) contents in serum were determined by using commercial kits (BioSino Bio-Technology and Science Inc., Beijing, China) on a fully automatic biochemical analyzer (BSI, Milano, Italy). Urea nitrogen (BUN) levels in allantoic fluid and nitric oxide synthase (NOS) levels in serum were determined by using a commercial kit purchased from Nanjing Jiancheng Bioengineering Institute (China) and tested on an automatic enzyme marker (Tecan, Grödig, Austria). Growth hormone (GH) and insulin (INS) contents in serum were determined by using an ELISA kit purchased from Rapidbio Company (Plymouth, MI, USA) and a Multifunctional Enzyme Marker (Tecan GENios, Männedorf, Switzerland).

### 2.4. Statistical Analysis

The experimental data were analyzed by using a proc mixed statistical analysis system based on the mixed effect model in SAS software (version 9.0; SAS Institute Inc., Cary, NC, USA). Dietary energy level and arginine level were fixed, and blocks were random.

The following model was used to analyze these data:

Y_ijk_ = μ + α_i_ + β_j_ + (αβ)_ij_ + P_k_ + ε_ijk_.

Y_ijk_ is the value of the individual sample from each replicate; μ is the overall mean; α_i_ is the dietary energy effect; β_j_ is the dietary arginine effect; (αβ)_ij_ is the interaction between dietary energy and arginine; P_k_ is the effect of block, and ε_ijk_ is the error component. When a significant interaction between the main effects was observed, Tukey–Kramer’s HSD test was used to compare the differences among the groups. A value of *p* ≤ 0.05 was considered significant.

## 3. Results

### 3.1. Embryo Growth and Development

There were no significant effects of arginine and interactions between energy and arginine on egg weight and embryo weight (Table 2 and Table 3). As shown in Table 2, eggs from birds fed low-energy diets and moderate diets were heavier than those fed high-energy diets at E0 and E11 (*p* < 0.05). The weight of eggs at E13 from the high-energy groups was significantly lower than that from the low-energy groups (*p* < 0.05). Compared with the low-energy and the moderate-energy groups, the weight of embryos at E13 and E15 in the high-energy groups was lower. (Table 3; *p* < 0.05). There were no significant effects of interactions between energy and arginine intake on eggshell weights during the incubation (Table 4).

As shown in Table 5, there was a significant effect of interaction between energy and arginine intake on the breast rate of embryos at E11, E13 or E17 (*p* < 0.01). Arginine supplementation in maternal diets significantly decreased the breast rate of embryos in high-energy groups at E11 and E13 and in low-energy groups at E17, but increased in low-energy groups at E13 and did not affect other energy groups. Compared with the moderate-energy diets, the maternal low-energy and high-energy diets during the laying period decreased the breast rate of embryos at E13, respectively (*p* < 0.01). Compared with the low-energy group, the high-energy group had a higher breast rate of embryos at E13, E15 and E21 (*p* < 0.01). Arginine supplementation in maternal diets significantly increased the breast rate of embryos at E11 *(p* < 0.01). 

As shown in Table 6, there was a significant effect of interaction between energy and arginine intake on the thigh rate of embryos at E19 or E21 (*p* < 0.05). Arginine supplementation decreased the thigh rate of embryos in high-energy groups at E19 or E21 and moderate-energy groups at E19 but did not affect other energy groups. The thigh rate of embryos in high-energy groups was lower than that in the low-energy or moderate-energy groups at E13, E15, E17, E19 or E21 (*p* < 0.01). Arginine supplementation significantly decreased the thigh rate of embryos at E19 (*p* < 0.01).

As shown in Table 7, the liver rate of embryos in high-energy groups was lower than that in moderate- or low-energy groups at E15, E17, E19 or E21 (*p* < 0.01), but was higher than that in low-energy groups at E11. There was a significant effect of interaction between energy and arginine intake of hens on the liver rate of embryos at E11 or E19. Arginine supplementation significantly increased the liver rate of embryos in low-energy groups at E11, but there was no effect of maternal energy intake on it in other energy groups. Arginine supplementation in the maternal diet increased the difference in liver rate of embryos at E19 between the moderate-energy group and high-energy group.

### 3.2. Biochemical and Endocrine Indexes of Embryonic Blood

As shown in Table 8, there was no significant effect of interaction between energy and arginine intake on the serum cholesterol (CHO) levels of embryos (*p* > 0.05). Compared with the low-energy diets, the maternal high-energy diets decreased the serum CHO level at E13, E15, E17 or E19 (*p* < 0.05). Arginine supplementation in maternal diets significantly decreased the serum CHO levels of embryos at E11 (*p* < 0.01).

As shown in Table 9, there was a significant effect of interaction between energy and arginine intake on the embryonic serum high density lipoprotein (HDL) content at E21 (*p* < 0.05). Arginine supplementation in maternal diets significantly increased the serum HDL level in low-energy groups at E21 but did not affect other energy groups. High energy supplied in maternal diets significantly decreased the serum HDL level in the high-arginine group at E21 (*p* < 0.05) and low energy supplied in maternal diets significantly increased the index in the high-arginine group (*p* < 0.05). Compared with the low-energy group, high energy supplied in maternal diets decreased the serum HDL level at E13, E15 or E21 (*p* < 0.05).

There were no significant effects of interactions between energy and arginine intake on the serum low-density lipoprotein (LDL) levels of embryos during the hatching period (See Table 10). Compared with the low-energy groups, the high-energy groups had lower embryonic serum LDL levels at E15, E19 or E21 (*p* < 0.01).

There were no significant effects of interactions between energy and arginine intake on the serum triglyceride (TG) levels of embryos during the hatching period (See Table 11). Compared with the low-energy diets, the maternal moderate-energy and high-energy diets during the laying period increased the embryonic serum TG level at E19 or E21 (*p* < 0.05).

Arginine supplementation significantly decreased the serum blood glucose (GLU) content of embryos at E11 (*p* < 0.01; See Table 12). With the increase in maternal dietary energy level, embryonic serum GLU level decreased significantly (*p* < 0.01). There were no significant effects of interactions between energy and arginine intake on the serum GLU levels of embryos during the hatching period.

As shown in Table 13, there were no significant effects of interactions between energy and arginine intake on the serum nitric oxide synthase (NOS) levels of embryos during the hatching period. High maternal energy groups had higher serum NOS levels of embryos at E11, E15 or E19 (*p* < 0.01). Arginine supplementation significantly increased the embryonic serum NOS level at E11, E15 or E19 (*p* < 0.01).

There was a significant effect of interaction between maternal energy and arginine intake on the serum growth hormone (GH) levels of embryos at E17 (*p* < 0.01, Table 14). High energy supplementation in maternal diets significantly increased the serum insulin (INS) levels of embryos at E13 (*p* < 0.05, Table 15).

As shown in Table 16, arginine supplementation in maternal diets during the laying period significantly decreased the urea nitrogen level in allantoic fluid at E11, E13 or E15 (*p* < 0.01). There were no effects of energy and interactions about energy and arginine intake on the urea nitrogen level in allantoic fluid from E11 to E17.

## 4. Discussion

The nutritional requirements for the embryonic development of birds come from eggs. The egg weight was positively correlated with the hatch weight of the chicks [31]. Granghelli et al. reported that maternal dietary ME levels had no effect on egg weight [32]. However, the study showed low-density diets (8.20 MJ ME/kg) improved laying performance and increased egg weight [33]. Li et al. reported that the dietary energy concentration of 9.36 MJ/kg significantly reduced the egg weight and embryo weight compared with 11.70 MJ/kg [34]. The egg weight resulting from high-energy diet intake was lower than that of other treatments at E0 in this research. We also found that the effects of energy in the maternal diet on egg weight gradually diminished with the incubation process.

In embryonic development, the relative weight of each organ can reflect the condition of embryo growth and development. Zhang et al. reported that restriction of maternal feed intake increased liver weight at E15, breast muscle weight at E19 and thigh weight at E17 [35]. Jiang et al. reported that maternal rations with 80% energy restriction significantly increased pectoral muscle weight and liver weight at E13 and E21 and leg muscle weight at E17 and E21 [36]. In the present study, maternal energy limitation could affect the breast rate of embryos at E13, E15 or E21, the thigh rate of embryos at E13, E15, E17, E19 or E21, and the liver rate of embryos at E11, E15, E17, E19 or E21. Our results are consistent with the above reports. In addition, we also found that maternal diet supplementation with 1.45% digestible arginine reduced the embryonic breast rate at E11 and thigh rate at E19. This may be related to the increase in the protein conversion rate caused by arginine. Arginine plays an important role in promoting muscle protein synthesis [37,38]. Interestingly, we found a significant effect of interaction between energy and arginine intake on the embryonic breast rate at E11, E13 or E17, and a significant effect of interaction between energy and arginine intake on the embryonic thigh rate at E19 or E21. This indicated that arginine could interfere with the impact of maternal energy levels on the developmental process of offspring embryos.

As an important indicator of lipid metabolism, cholesterol (CHO) is present in the blood as lipoproteins including LDL-CHO, HDL-CHO, and VLDL-CHO. HDL is synthesized by the liver, and LDL, a carrier of CHO, is produced by the metabolism of lipoproteins rich in TG in the blood [39]. Increasing LDL content will increase the absorption and metabolism of adipose tissue and promote animal growth. Serum HDL can be combined with excess blood lipids to transport CHO from surrounding tissues to the liver for decomposition. Li et al. reported that maternal energy restriction decreased the crude fat, CHO and energy levels of yolks during embryonic development, especially during the last week, but increased the serum HDL and LDL level [33]. The embryonic serum LDL level at E15, E19 and E21 decreased as the maternal dietary energy level increased, and the embryonic serum HDL level at E13, E15 and E21 decreased. Meanwhile, the serum CHO contents of the embryos in the high-energy diet treatment were significantly lower than those of the others at E13, E15, E17, and E19. These findings implied that a maternal low-energy diet promotes embryonic fat mobilization in offspring. HDL is a universal plasma-accepted lipoprotein used not only for CHO efflux from peripheral tissues but also for CHO excretion from the liver [40]. Lima found that the addition of arginine to the diet reduced serum triacylglycerol and cholesterol [41]. Fouad also found that the addition of arginine to broiler diets reduced the expression of genes encoding the liver enzymes FAS and HMG-CoA, which are involved in the biosynthesis of triacylglycerol and CHO [42]. CHO in embryos was reduced because of the maternal dietary supply of arginine at E11. But maternal arginine levels had no significant effect on embryonic serum HDL content. Consistent with the results of embryonic development, an interactive effect of maternal energy and arginine levels on embryonic serum HDL content was found at E21 in this trial.

Triglycerides (TGs) are mainly derived from the diet or liver synthesis. Serum TGs are closely related to the energy metabolism of the body and reflect the utilization of fat [43]. TGs are transported through the blood in the form of lipoproteins. Therefore, fat deposition in poultry mainly depends on the level of TGs in plasma [44]. In our experiment, the maternal high-energy diet significantly increased the serum TG level of the embryos at E19 and E21, which demonstrated that the maternal low-energy diet attenuated the fat metabolism in embryos at E19 and E21.

Blood glucose (GLU), as a source of energy in the body, releases energy through glycogen decomposition, which in turn provides a carbon framework for the synthesis of lipids to form cholesterol. GLU in the blood of broilers could effectively alleviate the effect caused by insufficient energy intake [45,46], which is called the GLU homeostasis mechanism [47]. INS reduces blood sugar by promoting the synthesis of sugar from GLU. In this experiment, the serum GLU content of E13 embryos in the low-energy group was significantly higher than that in the high-energy group. Meanwhile, the insulin (INS) content of embryos was consistent with the results for GLU content, and the INS content of embryos in the high-energy group was significantly higher than that in the low-energy group. The study showed that maternal dietary restriction increased serum GLU levels in 28-day-old offspring [34]. These results do seem to be correlated. Arginine stimulates the release of pancreatic hormones [48]. Pancreatic hormones include glucagon, INS, and GH. Kim reported that dietary supplementation with 0.4% arginine increased plasma concentrations of INS and GH by 24–27% compared to controls [49]. Xu et al. found higher serum concentrations of GH, IGF-1, and INS in broilers fed sufficient arginine than in those fed insufficient arginine [50]. In the present experiment, we found that the addition of 1.45% digestible arginine to the maternal diet significantly reduced serum GLU levels in embryos at E11. Pablo et al. found that INS appeared in chick embryos before β-cell recognizability and even in egg components before fertilization [51]. Therefore, we conjectured that the reduced GLU content in embryos was due to the action of arginine on the mother leading to maternal INS secretion and the deposition of INS in the egg, resulting in reduced serum GLU content in embryos.

Nitric oxide (NO) plays an important role in the non-specific immune response of killing microorganisms in the body [52]. The synthesis of NO is catalyzed by nitric oxide synthase (NOS) [53]. Arginine regulates the body’s immunity by stimulating NOS to produce NO [54,55]. In our research, the addition of a high dose of arginine in the maternal diet significantly increased the NOS level in the serum of the embryos. At the same time, a high level of ME significantly increased the serum NOS levels in the embryos. NO exerts its diastolic effect through the arginine-NO-cyclic adenosine monophosphate (L-Arg-NO-cAMP) pathway. NO has an important role in the diastolic response. The regulation of pulmonary artery pressure in broiler chickens is closely related to NO production [56]. Some studies found that hypoxia inhibited NOS activity and gene expression and reduced endogenous NO release [57]. In vivo studies also found that chronic hypoxia reduced pulmonary artery eNOS activity [58]. Early-term feeding could reduce the growth rate of broilers, thus alleviating the hypoxic state of the organism and thus alleviating problems such as pulmonary hypertension [59]. Huang et al. found that restriction of feeding resulted in lower plasma NO levels in broilers [60]. In the present study, lower embryonic serum NOS levels were found in the low maternal energy group at E11 and E19. This might be due to a decrease in embryonic intra-serum NOS activity because of maternal energy restriction, which slowed embryonic development, leading to problems related to hypoxia.

Studies in birds have shown that restricted feeding can alter plasma hormone levels that regulate growth and energy metabolism [61]. The main physiological role of growth hormone (GH) is to promote protein synthesis and stimulate the growth of bone joints and cartilage joints [62]. GH affects energy metabolism by inhibiting GLU use in muscle and tissue and promoting GLU production, thereby increasing blood GLU levels. At the same time, it also enhances lipolysis and reduces fat deposition [63]. In this experiment, there was a significant interaction effect of maternal energy and arginine level on embryonic serum GH level at E17. Neither high-energy and high-arginine treatment nor low-energy and low-arginine treatment in maternal diets was beneficial for serum GH levels in embryos.

Arginine is widely involved in the metabolism of animal organisms, such as regulating protein synthesis and promoting protein deposition. Emadi reported that arginine in the diet of broiler chickens improved growth performance and plasma parameters, especially albumin concentration [64]. Meanwhile, Yu et al. found that feeding arginine in broilers from 3 to 21 days after hatching resulted in improving plasma TP and albumin concentrations [48]. Urea nitrogen indicates protein metabolism. The lower the urea nitrogen content, the higher the protein utilization [65]. In the present study, arginine supplementation in the diets of laying hens significantly reduced urea nitrogen levels in the allantoic fluid of embryos at E11, E13 or E15. Allantoic fluid is the liquid secreted by the embryonic chicken embryo and stored in the allantoic cavity. The allantoic fluid contains urea and creatinine. Birds have an incomplete urea cycle and cannot synthesize arginine efficiently [66]. To meet the physiological needs of broilers, arginine is generally provided through a nutritional plan. The nutrition of the chicken embryo mainly comes from the egg yolk, which comes from the lipid and protein deposition of the hen from the liver to the ovary [67]. Therefore, it is presumed from the results that it is because the hens consume arginine to improve the urea cycle, which is then passed on to the embryo through the yolk. At the same time, the data showed that urea nitrogen gradually increased with the increase in embryo age, which was caused by the gradual maturity of the chicken embryo and the gradual decrease in the nutrient intake in the egg yolk.

## 5. Conclusions

Maternal restricted feeding improved embryonic development and regulated lipid metabolism-related indices in embryonic serum. Maternal dietary addition of digestible arginine had a significant effect on lipid metabolism indices in embryos. In summary, there is a maternal effect of maternal dietary energy and arginine levels on embryonic growth and development. The deposition of maternal nutrients affects the development of embryos. Furthermore, there is a reciprocal effect between energy and arginine, and arginine regulates embryonic developmental retardation due to insufficient maternal energy intake. Therefore, appropriate restriction of maternal feeding or dietary supplementation with arginine can be an effective way to regulate embryonic development and lipid metabolism.

## Figures and Tables

**Table 1:** Composition and nutrient levels of diets for broiler breeders during the laying period (air dry basis).

Item	Treatments					
	T1	T2	T3	T4	T5	T6
Ingredient						
Corn (g/kg)	495	492.5	640	636.8	651	647.8
Soybean meal (g/kg)	178.6	177.7	240	238.8	83.4	83
Corn protein powder (g/kg)	-	-	-	-	85.7	85.3
Wheat bran (g/kg)	225.4	224.3	-	-	-	-
Soybean oil (g/kg)	-	-	20	19.9	71.2	70.8
DL-Methionine (g/kg)	1.1	1.1	0.8	0.8	0.4	0.4
L-Lysine HCl (g/kg)	0.7	0.7	-	-	3.6	3.6
L-Arginine (g/kg)	0.5	5.5	-	5	3.5	8.5
Limestone (g/kg)	76.4	76	75.9	75.5	75.9	75.5
CaHPO_4_ (g/kg)	15	14.9	16	15.9	18	17.9
Salt (g/kg)	3	3	3	3	3	3
Choline chloride (g/kg)	1	1	1	1	1	1
Vitamin-mineral premix ^1^ (g/kg)	3.3	3.3	3.3	3.3	3.3	3.3
Total	1000	1000	1000	1000	1000	1000
Nutrient composition ^2^						
ME (MJ/kg)	10.08	10.08	11.88	11.88	13.68	13.68
Cross energy (MJ/kg)	(14.18)	(14.23)	(16.82)	(16.74)	(19.33)	(19.21)
CP (g/kg)	160.9 (160.5)	160.1 (159.7)	160.6 (160.1)	159.8 (159.7)	160.6 (160.3)	159.8 (159.8)
Digestible lysine (g/kg)	6.7	6.6	6.7	6.7	6.7	6.6
Digestible methionine (g/kg)	2.9	2.9	2.9	2.9	2.9	2.9
Digestible arginine (g/kg)	9.6	14.5	9.6	14.5	9.6	14.5
Arginine (g/kg)	(10.62)	(16.09)	(10.67)	(16.11)	(10.58)	(16.05)
Digestible methionine + cysteine (g/kg)	4.8	4.8	4.9	4.9	4.8	4.8
Ca (g/kg)	31.6 (31.5)	31.4 (31.4)	31.6 (31.5)	31.4 (31.3)	31.6 (31.5)	31.4 (31.4)
Total P (g/kg)	6.9 (6.8)	6.9 (6.9)	5.8 (5.7)	5.7 (5.8)	5.5 (5.5)	5.5 (5.4)
Available P (g/kg)	4.1	4.1	4.1	4.1	4.1	4
NaCl (g/kg)	2.9	2.9	2.9	2.9	2.9	2.9

^1^ The premix provided the following per kg of diets: VA 12,000 IU, VD 2400 IU, VE 30 IU, VK3 1.5 mg, VB12 0.012 mg, VB1 2.0 mg, biotin 0.20 mg, folic acid 1.2 mg, nicotinic acid 35 mg, pantothenic acid 12 mg, pyridoxine 4.5 mg, riboflavin 9 mg, Cu 8 mg, I 1.0 mg, Fe 80 mg, Mn 100 mg, Se 0.30 mg, Zn 80 mg. ^2^ The values in parentheses indicate the analyzed value. Others were calculated from data provided by Feed Database in China (2004) [28].

**Table 2:** Effects of maternal dietary energy and arginine level on the egg weight at different embryonic days.

Items	Energy			Arginine		Energy × Arginine						Pooled SEM	*p* Value		
Energy (MJ/kg)	10.08	11.88	13.68			10.08	10.08	11.88	11.88	13.68	13.68		Energy	Arginine	Energy × Arginine
Arginine (g/kg)				9.60	14.50	9.60	14.50	9.60	14.50	9.60	14.50				
E0 (g)	69.66 ^a^	69.12 ^a^	67.57 ^b^	68.48	69.08	69.72	69.59	68.63	69.60	67.09	68.04	0.5912	<0.0001	0.0824	0.3198
E11 (g)	64.76 ^a^	63.65 ^a^	61.72 ^b^	63.19	63.56	64.78	64.73	63.46	63.84	61.34	62.11	1.1408	0.0020	0.5815	0.8781
E13 (g)	63.91 ^a^	62.67 ^ab^	61.51 ^b^	62.78	62.62	63.38	64.45	63.70	61.64	61.25	61.77	1.0249	0.0084	0.7965	0.0846
E15 (g)	62.02	61.46	60.13	60.77	61.63	61.17	62.87	61.71	61.20	59.44	60.83	1.8710	0.3476	0.4286	0.6650
E17 (g)	61.07	60.19	59.54	60.05	60.49	60.69	61.45	60.11	60.26	59.34	59.75	1.3888	0.3003	0.5869	0.9535
E19 (g)	60.41	60.11	59.29	59.82	60.05	60.23	60.58	60.01	60.22	59.22	59.35	1.3143	0.4619	0.7616	0.9926

^a,b^ Means within a row with no common superscripts differ significantly (*p* < 0.05).

**Table 3:** Effects of maternal dietary energy and arginine level on the weight of embryos during the hatching period.

Items	Energy			Arginine		Energy × Arginine						Pooled SEM	*p* Value		
Energy (MJ/kg)	10.08	11.88	13.68			10.08	10.08	11.88	11.88	13.68	13.68		Energy	Arginine	Energy × Arginine
Arginine (g/kg)				9.60	14.50	9.60	14.50	9.60	14.50	9.60	14.50				
E11 (g)	3.71	3.94	3.68	3.73	3.83	3.61	3.81	3.91	3.96	3.66	3.71	0.168	0.0729	0.3078	0.7822
E13 (g)	8.35 ^a^	8.56 ^a^	7.61 ^b^	8.05	8.3	8.04	8.66	8.55	8.58	7.56	7.66	0.2836	<0.0001	0.1364	0.2927
E15 (g)	15.26 ^a^	16.06 ^a^	13.18 ^b^	14.68	14.98	14.69	15.82	16.43	15.68	12.92	13.44	0.6129	<0.0001	0.397	0.0938
E17 (g)	23.5	24.02	23.32	23.44	23.79	23.08	23.91	24.08	23.95	23.14	23.5	0.814	0.4579	0.4575	0.7098
E19 (g)	32.48	33.34	32.26	32.68	32.7	32.69	32.26	33.15	33.52	32.19	32.33	0.8003	0.1399	0.9538	0.7671
E21 (g)	48.98	49.49	48.53	48.75	49.25	48.83	49.14	49.09	49.89	48.35	48.71	1.3317	0.5995	0.5229	0.9611

^a,b^ Means within a row with no common superscripts differ significantly (*p* < 0.05).

**Table 4:** Effects of maternal dietary energy and arginine level on the eggshell weight during the hatching period.

Items	Energy			Arginine		Energy × Arginine						Pooled SEM	*p* Value		
Energy (MJ/kg)	10.08	11.88	13.68			10.08	10.08	11.88	11.88	13.68	13.68		Energy	Arginine	Energy × Arginine
Arginine (g/kg)				9.60	14.50	9.60	14.50	9.60	14.50	9.60	14.50				
E11 (g)	6.65	6.67	6.23	6.58	6.45	6.70	6.60	6.80	6.54	6.24	6.22	0.2868	0.0552	0.4441	0.8197
E13 (g)	6.54	6.24	6.19	6.44	6.22	6.66	6.43	6.38	6.11	6.27	6.12	0.2315	0.0755	0.1098	0.9314
E15 (g)	6.59	6.25	6.25	6.36	6.37	6.60	6.58	6.24	6.25	6.24	6.27	0.2369	0.0701	0.9626	0.9944
E17 (g)	6.19	6.23	6.11	6.22	6.13	6.26	6.12	6.29	6.17	6.12	6.10	0.1421	0.4815	0.2661	0.8011
E19 (g)	6.21	6.17	6.10	6.22	6.09	6.26	6.17	6.25	6.08	6.17	6.03	0.1892	0.6956	0.2318	0.9609

**Table 5:** Effects of maternal dietary energy and arginine level on the breast rate of embryos during the hatching period.

Items	Energy			Arginine		Energy × Arginine						Pooled SEM	*p* Value		
Energy (MJ/kg)	10.08	11.88	13.68			10.08	10.08	11.88	11.88	13.68	13.68		Energy	Arginine	Energy × Arginine
Arginine (g/kg)				9.60	14.50	9.60	14.50	9.60	14.50	9.60	14.50				
E11 (%)	2.84	2.85	2.69	2.96 ^b^	2.62 ^a^	2.78 ^ab^	2.91 ^ab^	3.10 ^a^	2.59 ^ab^	3.00 ^a^	2.37 ^b^	0.1991	0.4317	0.0055	0.0203
E13 (%)	3.76 ^b^	4.09 ^a^	3.24 ^c^	3.70	3.70	3.51 ^b^	4.02 ^a^	4.12 ^a^	4.06 ^a^	3.48 ^b^	3.01 ^b^	0.1664	<0.0001	0.9649	0.0008
E15 (%)	4.46 ^a^	4.44 ^a^	3.86 ^b^	4.23	4.27	4.48	4.44	4.32	4.56	3.91	3.82	0.1553	<0.0001	0.6792	0.2792
E17 (%)	3.45	3.40	3.54	3.48	3.44	3.58 ^a^	3.32 ^b^	3.40 ^ab^	3.39 ^ab^	3.47 ^ab^	3.62 ^a^	0.0970	0.1231	0.4943	0.0181
E19 (%)	3.59	3.52	3.54	3.60	3.50	3.58	3.60	3.54	3.49	3.67	3.40	0.1040	0.6069	0.1091	0.1549
E21 (%)	2.14 ^a^	2.01 ^b^	1.90 ^b^	2.02	2.01	2.07	2.22	2.08	1.93	1.91	1.88	0.0856	0.0010	0.9121	0.0528

^a–c^ Means within a row with no common superscripts differ significantly (*p* < 0.05).

**Table 6:** Effects of maternal dietary energy and arginine level on the thigh rate of embryos during the hatching period.

Items	Energy			Arginine		Energy × Arginine						Pooled SEM	*p* Value		
Energy (MJ/kg)	10.08	11.88	13.68			10.08	10.08	11.88	11.88	13.68	13.68		Energy	Arginine	Energy × Arginine
Arginine (g/kg)				9.60	14.50	9.60	14.50	9.60	14.50	9.60	14.50				
E11 (%)	13.41	13.03	12.70	13.23	12.86	13.64	13.19	13.30	12.75	12.75	12.64	0.4276	0.0731	0.1426	0.7522
E13 (%)	11.99 ^a^	11.43 ^a^	10.23 ^b^	11.09	11.34	11.95	12.04	11.18	11.69	10.14	10.31	0.3999	<0.0001	0.2741	0.7301
E15 (%)	9.83 ^a^	9.86 ^a^	8.96 ^b^	9.57	9.53	9.76	9.90	10.02	9.70	8.94	8.98	0.2539	<0.0001	0.7572	0.4100
E17 (%)	9.70 ^a^	9.58 ^a^	8.21 ^b^	9.09	9.24	9.62	9.79	9.62	9.54	8.02	8.40	0.2483	<0.0001	0.2834	0.4465
E19 (%)	11.73 ^a^	11.03 ^b^	10.03 ^c^	11.36 ^a^	10.50 ^b^	11.43 ^ab^	12.03 ^a^	11.43 ^ab^	10.64 ^b^	11.22 ^ab^	8.84 ^c^	0.3220	<0.0001	<0.0001	<0.0001
E21 (%)	10.99 ^a^	10.73 ^a^	8.60 ^b^	10.21	10.00	10.60 ^a^	11.38 ^a^	10.90 ^a^	10.56 ^ab^	9.13 ^bc^	8.07 ^c^	0.4864	<0.0001	0.4564	0.0350

^a–c^ Means within a row with no common superscripts differ significantly (*p* < 0.05).

**Table 7:** Effects of maternal dietary energy and arginine level on the liver rate of embryos during the hatching period.

Items	Energy			Arginine		Energy × Arginine						Pooled SEM	*p* Value		
Energy (MJ/kg)	10.08	11.88	13.68			10.08	10.08	11.88	11.88	13.68	13.68		Energy	Arginine	Energy × Arginine
Arginine (g/kg)				9.60	14.50	9.60	14.50	9.60	14.50	9.60	14.50				
E11 (%)	1.20 ^b^	1.39 ^a^	1.38 ^a^	1.29	1.36	1.07 ^b^	1.33 ^ab^	1.35 ^a^	1.43 ^a^	1.44 ^a^	1.32 ^ab^	0.0836	0.0036	0.1296	0.0127
E13 (%)	1.61	1.51	1.48	1.54	1.52	1.60	1.62	1.51	1.51	1.53	1.42	0.0850	0.0983	0.5788	0.5261
E15 (%)	1.79 ^a^	1.80 ^a^	1.28 ^b^	1.64	1.61	1.79	1.79	1.86	1.74	1.27	1.30	0.0675	<0.0001	0.4908	0.2809
E17 (%)	2.01 ^a^	1.86 ^b^	1.55 ^c^	1.79	1.82	2.03	1.98	1.84	1.88	1.48	1.61	0.0636	<0.0001	0.2973	0.1620
E19 (%)	1.94 ^a^	1.89 ^a^	1.67 ^b^	1.83	1.84	1.91 ^a^	1.96 ^a^	1.82 ^ab^	1.96 ^a^	1.75 ^ab^	1.59 ^b^	0.0800	<0.0001	0.8546	0.0351
E21 (%)	1.58 ^a^	1.59 ^a^	1.21 ^b^	1.47	1.44	1.66	1.51	1.55	1.62	1.21	1.21	0.0863	<0.0001	0.5608	0.2030

^a–c^ Means within a row with no common superscripts differ significantly (*p* < 0.05).

**Table 8:** Effects of maternal dietary energy and arginine level on the serum CHO content of embryos.

Items	Energy			Arginine		Energy × Arginine						Pooled SEM	*p* Value		
Energy (MJ/kg)	10.08	11.88	13.68			10.08	10.08	11.88	11.88	13.68	13.68		Energy	Arginine	Energy × Arginine
Arginine (g/kg)				9.60	14.50	9.60	14.50	9.60	14.50	9.60	14.50				
E11 (mmol/L)	5.60	5.55	5.36	6.02 ^a^	4.98 ^b^	6.47	4.72	5.78	5.32	5.82	4.90	0.4846	0.7680	0.0008	0.1846
E13 (mmol/L)	5.12 ^a^	4.74 ^ab^	4.28 ^b^	4.70	4.73	4.91	5.33	5.03	4.45	4.15	4.41	0.4279	0.0322	0.8953	0.2263
E15 (mmol/L)	7.63 ^a^	7.38 ^a^	4.26 ^b^	6.61	6.23	7.98	7.28	7.54	7.22	4.33	4.19	0.5820	<0.0001	0.2654	0.7900
E17 (mmol/L)	13.02 ^a^	13.12 ^a^	10.83 ^b^	12.79	11.85	13.31	12.72	13.63	12.62	11.44	10.21	1.1002	0.0090	0.1496	0.9164
E19 (mmol/L)	12.35 ^a^	8.98 ^b^	8.32 ^b^	9.97	9.80	12.19	12.51	8.92	9.03	8.79	7.86	0.8579	<0.0001	0.7363	0.5486
E21 (mmol/L)	13.48	12.09	11.94	13.01	11.99	13.48	13.48	12.56	11.61	12.99	10.89	1.4196	0.2539	0.2237	0.5811

^a,b^ Means within a row with no common superscripts differ significantly (*p* < 0.05).

**Table 9:** Effects of maternal dietary energy and arginine level on the serum HDL content of embryos.

Items	Energy			Arginine		Energy × Arginine						Pooled SEM	*p* Value		
Energy (MJ/kg)	10.08	11.88	13.68			10.08	10.08	11.88	11.88	13.68	13.68		Energy	Arginine	Energy × Arginine
Arginine (g/kg)				9.60	14.50	9.60	14.50	9.60	14.50	9.60	14.50				
E11 (mmol/L)	0.12	0.12	0.08	0.10	0.11	0.10	0.13	0.12	0.12	0.09	0.08	0.03	0.2384	0.7846	0.7023
E13 (mmol/L)	0.15 ^a^	0.14 ^ab^	0.10 ^b^	0.12	0.14	0.14	0.17	0.13	0.14	0.10	0.10	0.03	0.0240	0.3171	0.7727
E15 (mmol/L)	0.29 ^a^	0.25 ^a^	0.14 ^b^	0.23	0.22	0.33	0.25	0.23	0.27	0.14	0.14	0.07	0.0104	0.7074	0.4904
E17 (mmol/L)	0.41 ^b^	0.73 ^a^	0.39 ^b^	0.48	0.54	0.36	0.46	0.71	0.75	0.37	0.41	0.10	<0.0001	0.3377	0.8902
E19 (mmol/L)	0.89	0.74	1.08	0.94	0.87	0.96	0.81	0.64	0.85	1.21	0.95	0.27	0.2216	0.6656	0.4294
E21 (mmol/L)	2.06 ^a^	1.82 ^a^	1.34 ^b^	1.65	1.82	1.72 ^ab^	2.39 ^a^	1.82 ^ab^	1.81 ^ab^	1.42 ^bc^	1.27 ^b^	0.23	0.0005	0.2120	0.0407

^a–c^ Means within a row with no common superscripts differ significantly (*p* < 0.05).

**Table 10:** Effects of maternal dietary energy and arginine level on the serum LDL content of embryos.

Items	Energy			Arginine		Energy × Arginine						Pooled SEM	*p* Value		
Energy (MJ/kg)	10.08	11.88	13.68			10.08	10.08	11.88	11.88	13.68	13.68		Energy	Arginine	Energy × Arginine
Arginine (g/kg)				9.60	14.50	9.60	14.50	9.60	14.50	9.60	14.50				
E11 (mmol/L)	1.98	1.94	1.95	2.06	1.85	2.08	1.89	2.06	1.83	2.05	1.85	0.3560	0.9856	0.3192	0.9954
E13 (mmol/L)	2.16	2.04	1.68	2.01	1.91	2.21	2.11	2.09	1.99	1.72	1.65	0.2728	0.0539	0.5542	0.9968
E15 (mmol/L)	4.24 ^a^	4.07 ^a^	2.98 ^b^	3.77	3.76	4.27	4.21	4.04	4.11	3.01	2.95	0.2901	<0.0001	0.9318	0.9313
E17 (mmol/L)	6.16	6.48	5.34	6.31	5.68	6.32	6.00	6.92	6.04	5.69	5.00	0.9457	0.2331	0.2606	0.9111
E19 (mmol/L)	4.48 ^a^	4.67 ^a^	3.05 ^b^	4.41	3.73	4.82	4.13	5.11	4.24	3.30	2.81	0.6542	0.0032	0.0818	0.9173
E21 (mmol/L)	3.28 ^a^	2.49 ^b^	1.59 ^c^	2.50	2.41	3.16	3.40	2.74	2.24	1.59	1.58	0.4420	<0.0001	0.7321	0.4921

^a–c^ Means within a row with no common superscripts differ significantly (*p* < 0.05).

**Table 11:** Effects of maternal dietary energy and arginine on the serum TG content of embryos.

Items	Energy			Arginine		Energy × Arginine						Pooled SEM	*p* Value		
Energy (MJ/kg)	10.08	11.88	13.68			10.08	10.08	11.88	11.88	13.68	13.68		Energy	Arginine	Energy × Arginine
Arginine (g/kg)				9.60	14.50	9.60	14.50	9.60	14.50	9.60	14.50				
E11 (mmol/L)	5.02	5.25	5.45	5.31	5.18	5.08	4.96	5.27	5.24	5.59	5.32	0.2511	0.0644	0.3591	0.7834
E13 (mmol/L)	4.95	5.09	5.35	5.22	5.04	5.00	4.90	5.26	4.93	5.39	5.31	0.3885	0.3443	0.4414	0.8803
E15 (mmol/L)	4.60	4.86	5.04	4.85	4.81	4.60	4.59	4.92	4.80	5.04	5.04	0.3548	0.2218	0.8255	0.9659
E17 (mmol/L)	3.92	4.03	4.12	4.05	4.00	3.92	3.92	4.05	4.01	4.17	4.07	0.2940	0.6479	0.7803	0.9691
E19 (mmol/L)	2.87 ^b^	3.31 ^a^	3.34 ^a^	3.19	3.15	2.88	2.86	3.33	3.28	3.36	3.32	0.2351	0.0156	0.8017	0.9955
E21 (mmol/L)	1.52 ^b^	1.81 ^a^	1.85 ^a^	1.75	1.70	1.54	1.51	1.81	1.80	1.90	1.80	0.1620	0.0177	0.6181	0.9089

^a,b^ Means within a row with no common superscripts differ significantly (*p* < 0.05).

**Table 12:** Effects of maternal dietary energy and arginine on the serum GLU content of embryos.

Items	Energy			Arginine		Energy × Arginine						Pooled SEM	*p* Value		
Energy (MJ/kg)	10.08	11.88	13.68			10.08	10.08	11.88	11.88	13.68	13.68		Energy	Arginine	Energy × Arginine
Arginine (g/kg)				9.60	14.50	9.60	14.50	9.60	14.50	9.60	14.50				
E11 (mmol/L)	9.86	9.49	9.16	9.98 ^a^	9.02 ^b^	10.62	9.11	9.96	9.03	9.37	8.94	0.5818	0.2478	0.0084	0.4362
E13 (mmol/L)	9.78 ^a^	8.73 ^b^	7.06 ^c^	8.78	8.27	9.85	9.71	9.38	8.09	7.12	7.01	0.6399	<0.0001	0.1745	0.3403
E15 (mmol/L)	9.77	9.61	9.51	9.73	9.53	9.96	9.57	9.65	9.56	9.57	9.46	0.5684	0.8147	0.5543	0.9141
E17 (mmol/L)	8.09	7.79	8.82	8.56	7.90	8.49	7.69	8.32	7.25	8.88	8.77	0.8952	0.2578	0.2107	0.7406
E19 (mmol/L)	9.24	8.62	8.69	8.98	8.72	9.51	8.98	8.67	8.57	8.75	8.62	0.6151	0.3112	0.4841	0.8616
E21 (mmol/L)	10.04	10.19	9.85	10.14	9.91	10.22	9.87	10.21	10.17	9.99	9.71	0.5813	0.7056	0.5025	0.9223

^a–c^ Means within a row with no common superscripts differ significantly (*p* < 0.05).

**Table 13:** Effects of maternal dietary energy and arginine on the serum NOS content of embryos.

Items	Energy			Arginine		Energy × Arginine						Pooled SEM	*p* Value		
Energy (MJ/kg)	10.08	11.88	13.68			10.08	10.08	11.88	11.88	13.68	13.68		Energy	Arginine	Energy × Arginine
Arginine (g/kg)				9.60	14.50	9.60	14.50	9.60	14.50	9.60	14.50				
E11 (U/mL)	5.61 ^c^	6.78 ^b^	7.46 ^a^	6.29 ^b^	6.94 ^a^	5.19	6.03	6.52	7.04	7.16	7.76	0.3073	<0.0001	0.0017	0.7558
E15 (U/mL)	9.46 ^b^	10.29 ^b^	12.87 ^a^	9.85 ^b^	11.90 ^a^	8.53	10.39	8.94	11.64	12.06	13.68	0.5691	<0.0001	<0.0001	0.4217
E19 (U/mL)	12.84 ^c^	13.61 ^b^	14.41 ^a^	13.18 ^b^	14.06 ^a^	12.42	13.25	13.18	14.04	13.94	14.88	0.4142	0.0003	0.0022	0.9831

^a–c^ Means within a row with no common superscripts differ significantly (*p* < 0.05).

**Table 14:** Effects of maternal dietary energy and arginine on the serum GH levels of embryos.

Items	Energy			Arginine		Energy × Arginine						Pooled SEM	*p* Value		
Energy (MJ/kg)	10.08	11.88	13.68			10.08	10.08	11.88	11.88	13.68	13.68		Energy	Arginine	Energy × Arginine
Arginine (g/kg)				9.60	14.50	9.60	14.50	9.60	14.50	9.60	14.50				
E13 (μg/L)	12.89	12.99	12.71	12.69	13.04	12.62	13.17	12.78	13.2	12.67	12.75	1.4821	0.9642	0.6891	0.9734
E17 (μg/L)	12.65	14.21	12.53	13.59	12.67	12.42 ^ab^	12.88 ^ab^	13.56 ^ab^	14.85 ^a^	14.79 ^a^	10.28 ^b^	1.147	0.0946	0.1815	0.0038
E21 (μg/L)	12.6	14.53	13.39	12.9	14.1	11.45	13.74	13.73	15.32	13.52	13.25	1.2467	0.1098	0.1076	0.338

^a,b^ Means within a row with no common superscripts differ significantly (*p* < 0.05).

**Table 15:** Effects of maternal dietary energy and arginine on the serum INS levels of embryos.

Items	Energy			Arginine		Energy × Arginine						Pooled SEM	*p* Value		
Energy (MJ/kg)	10.08	11.88	13.68			10.08	10.08	11.88	11.88	13.68	13.68		Energy	Arginine	Energy × Arginine
Arginine (g/kg)				9.60	14.50	9.60	14.50	9.60	14.50	9.60	14.50				
E13 (μg/L)	10.27 ^b^	11.48 ^b^	12.12 ^a^	10.94	11.64	9.8	10.73	11	11.96	12.01	12.23	0.9425	0.0318	0.2097	0.8233
E17 (μg/L)	10.75	11.01	12.09	11.09	11.48	10.48	11.01	10.82	11.21	11.97	12.21	0.8942	0.1049	0.4641	0.9744
E21 (μg/L)	11.41	11.6	12.2	11.99	11.49	11.09	11.74	11.27	11.93	12.11	12.29	0.8266	0.3873	0.3111	0.8955

^a,b^ Means within a row with no common superscripts differ significantly (*p* < 0.05).

**Table 16:** Effects of maternal dietary energy and arginine on the level of urea nitrogen in allantoic fluid during the hatching period.

Items	Energy			Arginine		Energy × Arginine						Pooled SEM	*p* Value		
Energy (MJ/kg)	10.08	11.88	13.68			10.08	10.08	11.88	11.88	13.68	13.68		Energy	Arginine	Energy × Arginine
Arginine (g/kg)				9.60	14.50	9.60	14.50	9.60	14.50	9.60	14.50				
E11 (mmol/L)	0.34	0.33	0.34	0.36 ^a^	0.32 ^b^	0.36	0.32	0.36	0.31	0.35	0.32	0.5818	0.8240	0.0001	0.7009
E13 (mmol/L)	0.53	0.54	0.53	0.55 ^a^	0.52 ^b^	0.55	0.52	0.57	0.51	0.54	0.52	0.0139	0.5887	0.0007	0.2407
E15 (mmol/L)	1.53	1.54	1.54	1.55 ^a^	1.52 ^b^	1.54	1.52	1.56	1.52	1.55	1.52	0.0146	0.7685	0.0061	0.9236
E17 (mmol/L)	2.39	2.35	2.40	2.41	2.35	2.44	2.34	2.36	2.34	2.44	2.35	0.0181	0.7441	0.2339	0.7650

^a,b^ Means within a row with no common superscripts differ significantly (*p* < 0.05).

## Data Availability

The data presented in this study are available in the present article and shared with consent and in accordance with all authors.

## References

[1] Surai P.F. (2000). Effect of selenium and vitamin E content of the maternal diet on the antioxidant system of the yolk and the developing chick. Br. Poult. Sci..

[2] Tona K., Onagbesan O., De Ketelaere B., Decuypere E., Bruggeman V. (2004). Effects of age of broiler breeders and egg storage on egg quality, hatchability, chick quality, chick weight, and chick posthatch growth to forty-two days. J. Appl. Poult. Res..

[3] Iqbal J., Mukhtar N., Rehman Z.U., Khan S.H., Ahmad T., Anjum M.S., Pasha R.H., Umar S. (2017). Effects of egg weight on the egg quality, chick quality, and broiler performance at the later stages of production (week 60) in broiler breeders. J. Appl. Poult. Res..

[4] Johnson-Dahl M.L., Zuidhof M.J., Korver D.R. (2017). The effect of maternal canthaxanthin supplementation and hen age on breeder performance, early chick traits, and indices of innate immune function. Poult. Sci..

[5] Li F., Ning H., Duan X., Chen Z., Xu L. (2021). Effect of dietary l-arginine of broiler breeder hens on embryonic development, apparent metabolism, and immunity of offspring. Domest. Anim. Endocrinol..

[6] Saunders-Blades J.L., Korver D.R. (2015). Effect of hen age and maternal vitamin D source on performance, hatchability, bone mineral density, and progeny in vitro early innate immune function. Poult. Sci..

[7] (2004). Nutrient Requirements of Swine.

[8] Holsheimer J.P., Veerkamp C.H. (1992). Effect of dietary energy, protein, and lysine content on performance and yields of two strains of male broiler chicks. Poult. Sci..

[9] Zhang M., Li Q., Liu P., Xu L., Su K., Wang D., Zhou R., Chen H. (2018). Study on Dietary Energy and Protein Requirements of Dawufen No.1 Commercial Layer Chicks Aged 5 to 9 Weeks. J. Anim. Nutr..

[10] Akinmoladun O.F., Falowo A.B. (2021). Energy manipulation of isonitrogenous diets for broiler chickens. S. Afr. J. Anim. Sci..

[11] Zuidhof M.J. (2018). Lifetime productivity of conventionally and precision-fed broiler breeders. Poult. Sci..

[12] Avila L.P., Sweeney K.M., Evans C.R., White D.L., Kim W.K., Regmi P., Williams S.M., Nicholds J., Wilson J.L. (2023). Body composition, gastrointestinal, and reproductive differences between broiler breeders fed using everyday or skip-a-day rearing programs. Poult. Sci..

[13] Heijmans J., Duijster M., Gerrits W.J., Kemp B., Kwakkel R.P., van den Brand H. (2021). Impact of growth curve and dietary energy-to-protein ratio on productive performance of broiler breeders. Poult. Sci..

[14] Kersten S. (2001). Mechanisms of nutritional and hormonal regulation of lipogenesis. EMBO Rep..

[15] Ginsberg H.N. (2002). New perspectives on atherogenesis: Role of abnormal triglyceride-rich lipoprotein metabolism. Circulation.

[16] Han G.P., Kim D.Y., Kim K.H., Kim J.H., Kil D.Y. (2023). Effect of dietary concentrations of metabolizable energy and neutral detergent fiber on productive performance, egg quality, fatty liver incidence, and hepatic fatty acid metabolism in aged laying hens. Poult. Sci..

[17] Pérez-Bonilla A., Novoa S., García J., Mohiti-Asli M., Frikha M., Mateos G.G. (2012). Effects of energy concentration of the diet on productive performance and egg quality of brown egg-laying hens differing in initial body weight. Poult. Sci..

[18] Castro F.L., Su S., Choi H., Koo E., Kim W.K. (2019). L-Arginine supplementation enhances growth performance, lean muscle, and bone density but not fat in broiler chickens. Poult. Sci..

[19] Uyanga V.A., Xin Q., Sun M., Zhao J., Wang X., Jiao H., Onagbesan O.M., Lin H. (2022). Research Note: Effects of dietary L-Arginine on the production performance and gene expression of reproductive hormones in laying hens fed low crude protein diets. Poult. Sci..

[20] Gao T., Zhao M.M., Zhang L., Li J.L., Yu L.L., Lv P.A., Gao F., Zhou G.H. (2017). Effects of in ovo feeding of l-arginine on the development of lymphoid organs and small intestinal immune barrier function in posthatch broilers. Anim. Feed. Sci. Technol..

[21] Esser A.F., Gonçalves D.R., Rorig A., Cristo A.B., Perini R., Fernandes J.I. (2017). Effects of guanidionoacetic acid and arginine supplementation to vegetable diets fed to broiler chickens subjected to heat stress before slaughter. Braz. J. Poult. Sci..

[22] Toghyani M., Tahmasebi S., Modaresi M., Fosoul S.S. (2019). Effect of Arginine and Threonine in Ovo Supplementation on Immune Responses and Some Serum Biochemical Attributes in Broiler Chickens. Ital. J. Anim. Sci..

[23] Jobgen W.S., Fried S.K., Fu W.J., Meininger C.J., Wu G. (2006). Regulatory role for the Arginine- nitric oxide pathway in metabolism of energy substrates. Nutr. Biochem..

[24] Jobgen W.S., Meininger C.J., Smith S.B., Spencer T.E., Jobgen S.C., Lee M.-J., Fried S.K., Wu G. (2007). Dietary Arginine supplementation reduces fat mass in diet- induced- obese rats by improving glucose and fatty acid metabolism. FASEB J..

[25] Wu G.Y., Lee M.J., Fried S.K. (2007). The Arginine- NO pathway modulates lipolysis in adipose tissues of obese human subjects. FASEB J..

[26] Yousif A.M.F. (2013). Effect of Dietary L-Arginine Supplementation on Growth Performance, Abdominal Lipid Rate, and Lipid Metabolism in Broiler Chicks. Ph.D. Thesis.

[27] Vaughn S.E. (2012). Review of the third edition of the Guide for the Care and Use of Agricultural Animals in Research and Teaching (Short Survey). J. Am. Assoc. Lab. Anim. Sci..

[28] (2004). Tables of feed ingredients and nutritional values in China, 15th edition, 2004. China Feed.

[29] Shan Y.L., Huang R.L. (2001). Modern Broiler Production Manual.

[30] Niu S., Xu L., Zhang H., Lu L., Lv R., Tian B. (2013). Effects of maternal dietary energy level on lipid metabolism related indexes in embryonic yolk and serum during the middle laying period in broiler breeders. Chin. J. Anim. Nutr..

[31] Pinchasov Y. (1991). Relationship between the weight of hatching eggs and subsequent early performance of broiler chicks. Br. Poult. Sci..

[32] Granghelli C.A., Burbarelli M.F.C., Lelis K.D., Pelissari P.H., Utimi N.B.P., Leite B.G.S., Roque F.A., Zorzetto P.S., Balieiro J.C.C., Araujo L.F. (2019). Effects of dietary metabolizable energy levels and beak trimming on the performance, egg quality, and economic viability of layers. Poult. Sci..

[33] Li F., Mou S.Y., Liu Y., Jiang D., Wang C., Chen Y.L., Ren H.L., Xu L.M. (2018). Maternal dietary energy levels affected the lipid deposition of offspring embryos at the end of the laying period of broiler breeder hens. Ital. J. Anim. Sci..

[34] Li F., Shan M.X., Gao X., Yang Y., Yang X., Zhang Y.Y., Hu J.W., Shan A.S., Cheng B.J. (2019). Effects of nutrition restriction of fat- and lean-line broiler breeder hens during the laying period on offspring performance, blood biochemical parameters, and hormone levels. Domest. Anim. Endocrinol..

[35] Zhang Y., Dan A., Li F., Hu J., Wang L., Li Y., Cheng B., Bi C. (2011). Effects of restricted feeding of hens on blood indicators related to lipid metabolism in offspring embryos. China Agric. Sci..

[36] Jiang D. (2014). Effect of Low-Energy Diets on Embryonic Fat Metabolism of Broiler Breeders in the Late Egg-Laying Period. Master’s Thesis.

[37] Laudadio V., Passantino L., Perillo A., Lopresti G., Passantino A., Khan R.U., Tufarelli V. (2012). Productive performance and histological features of intestinal mucosa of broiler chickens fed different dietary protein levels. Poult. Sci..

[38] Cao Y., Wang Q., Du Y., Liu F., Zhang Y., Feng Y., Jin F. (2016). L-Arginine and docetaxel syner-gistically enhance anti-tumor immunity by modifying the immune status of tumor-bearing mice. Int. Immunopharmacol..

[39] Thommes R.C., Shulman R.W. (1967). Endocrine control of lipid metabolism in the developing chick embryo. Gen. Comp. Endocrinol..

[40] Lewis G.F. (2005). New Insights Into the Regulation of HDL Metabolism and Reverse Cholesterol Transport. Circ. Res..

[41] Lima E.D., de Oliveira D.H., de Abreu M.L., Rosa P.V., de Laurentiz A.C., Naves L.D., Rodrigues P.B. (2021). Supplemental L-arginine improves feed conversion and modulates lipid metabolism in male and female broilers from 29 to 42 days of age. Animal.

[42] Fouad A.M., El-Senousey H.K., Yang X.J., Yao J.H. (2013). Dietary L-Arginine supplementation reduces abdominal fat content by modulating lipid metabolism in broiler chickens. Animal.

[43] Lee S., Lee K., Choi G., Desai M., Lee S., Pang M., Jo I., Kim Y. (2013). Feed restriction during pregnancy/lactation induces programmed changes in lipid, adiponectin and leptin levels with gender differences in rat offspring. J. Matern.-Fetal Neonatal Med..

[44] Griffin H.D., Whitehead C.C. (1982). Plasma lipoprotein concentration as an indicator of fatness in broilers: Development and use of a simple assay for plasma very low density lipoproteins. Br. Poult. Sci..

[45] Rajman M., Juráni M., Lamošová D., Máčajová M., Sedlačková M., Košťál Ľ., Ježová D., Výboh P. (2006). The effects of feed restriction on plasma biochemistry in growing meat type chickens (*Gallus gallus*). Comp. Biochem. Physiol. Part A Mol. Integr. Physiol..

[46] Sato M., Tachibana T., Furuse M. (2006). Total lipid and triacylglycerol contents in the liver of broiler and layer chickens at embryonic stages and hatching. Anim. Sci. J..

[47] Dewil E., Darras V.M., Spencer G.S., Lauterio T.J., Decuypere E. (1999). The regulation of GH-dependent hormones and enzymes after feed restriction in dwarf and control chickens. Life Sci..

[48] Yu L.L., Gao T., Zhao M.M., Lv P.A., Zhang L., Li J.L., Jiang Y., Gao F., Zhou G.H. (2018). In ovo feeding of L-Arginine alters energy metabolism in post-hatch broilers. Poult. Sci..

[49] Kim S.W., McPherson R.L., Wu G. (2004). Dietary Arginine Supplementation Enhances the Growth of Milk-Fed Young Pigs. J. Nutr..

[50] Xu Y.Q., Guo Y.W., Shi B.L., Yan S.M., Guo X.Y. (2018). Dietary Arginine supplementation enhances the growth performance and immune status of broiler chickens. Livest. Sci..

[51] Pablo F.D., Roth J., Hernandez E., Pruss R.M. (1982). Insulin Is Present in Chicken Eggs and Early Chick Embryos. Endocrinology.

[52] Moilanen E., Whittle B., Moncada S. (1999). Nitric Oxide as a factor in inflammation. Inflammation Basic Principles and Clinical Correlates.

[53] Mittal A., Kakkar R. (2020). Nitric Oxide Synthases and Their Inhibitors: A Review. Lett. Drug Des. Discov..

[54] Salil G., Nevin K.G., Rajamohan T. (2012). Arginine-rich coconut kernel diet influences nitric oxide synthase activity in alloxandiabetic rats. J. Sci. Food Agric..

[55] Luo H.J., Yang Z., Zeng J., Luo X.C., Liang K. (2006). Effect of l-Arginine on serum no, nos and thymus t cells subsets under heat stress in mice. Acta Nutr. Sin..

[56] Wang J., Wang X., Xiang R., Sun W. (2002). Effect of L-NAME on pulmonary arterial pressure, plasma nitric oxide and pulmonary hypertension syndrome morbidity in broilers. Br. Poult. Sci..

[57] McQuillan L.P., Leung G.K., Marsden P.A., Kostyk S.K., Kourembanas S. (1994). Hypoxia inhibits expression of eNOS via transcriptional and posttranscriptional mechanisms. Am. J. Physiol.-Heart Circ. Physiol..

[58] Berkenbosch J.W., Baribeau J., Perreault T. (2000). Decreased synthesis and vasodilation to nitric oxide in piglets with hypoxia-induced pulmonary hypertension. Am. J. Physiol.-Lung Cell. Mol. Physiol..

[59] Reeves J.T., Ballam G., Hofmeister S., Pickett C., Morris K., Peacock A. (1991). Improved arterial oxygenation with feed restriction in rapidly growing broiler chickens. Comp. Biochem. Physiol. A Comp. Physiol..

[60] Huang Y., Pan J., Tang Z., Li J., Tan X., Wang X. (2007). Effects of early and late feeding on plasma NO levels, erythrocyte specific volume and cardiac index in broiler chickens. Livest. Vet. Med..

[61] Buyse J., Decuypere E., Darras V.M., Vleurick L.M., Kuhn E.R., Veldhuis J.D. (2000). Food deprivation and feeding of broiler chickens is associated with rapid and interdependent changes in the somatotrophic and thyrotrophic axes. Br. Poult. Sci..

[62] Kim J.W. (2010). The endocrine regulation of chicken growth. Asian-Australas. J. Anim. Sci..

[63] Zhang Y., Shan A., Li F., Hu J., Wang L., Li Y., Cheng B., Bi C. (2011). Effect of Maternal Feed Restriction on Serum Lipid Metabolism During Embryo Period. Sci. Agric. Sin..

[64] Emadi M., Kaveh K., Bejo M.H., Ideris A., Jahanshiri F., Ivan M., Alimon R.A. (2012). Growth Performance and Blood Parameters as Influenced by Different Levels of Dietary Arginine in Broiler Chickens. J. Anim. Vet. Adv..

[65] Chikhou F.H., Moloney A.P., Allen P., Quirke J.F., Austin F.H., Roche J.F. (1993). Long-term effects of cimaterol in Friesi an steers: I, Growth, feed efficiency, and selected carcass traits. J. Anim. Sci..

[66] Oliveira C.H., Dias K.M., Bernardes R.D., Diana T.F., Rodrigueiro R.J., Calderano A.A., Albino L.F. (2022). The effects of Arginine supplementation through different ratios of Arginine: Lysine on performance, skin quality and creatine levels of broiler chickens fed diets reduced in protein content. Poult. Sci..

[67] Vieira P.M., Vieira A.V., Sanders E.J., Steyrer E., Nimpf J., Schneider W.J. (1995). Chicken yolk contains bona fide high density lipoprotein particles. J. Lipid Res..

